# MicroRNA 219-5p inhibits alveolarization by reducing platelet derived growth factor receptor-alpha

**DOI:** 10.1186/s12931-021-01654-7

**Published:** 2021-02-17

**Authors:** Amelia Freeman, Luhua Qiao, Nelida Olave, Gabriel Rezonzew, Samuel Gentle, Brian Halloran, Gloria S. Pryhuber, Amit Gaggar, Trent E. Tipple, Namasivayam Ambalavanan, Charitharth Vivek Lal

**Affiliations:** 1 Division of Neonatology, Department of Pediatrics, Women and Infants Center, University of Alabama At Birmingham, 176F Suite 9380619 South 19th Street, Birmingham, AL 35249-7335 USA; 2 Program in Matrix and Pulmonary Biology, Department of Medicine, University of Alabama, Birmingham, AL USA; 3 Department of Pediatrics, University of Rochester Medical Center, Rochester, NY USA; 4 Center for Pregnancy and Newborn Research, Section of Neonatal-Perinatal Medicine, University of Oklahoma College of Medicine, Oklahoma, OK USA

**Keywords:** Lung development, Infant, Bronchopulmonary dysplasia, microRNAs

## Abstract

**Background:**

MicroRNA (miR) are small conserved RNA that regulate gene expression post-transcription. Previous genome-wide analysis studies in preterm infants indicate that pathways of miR 219-5p are important in infants with Bronchopulmonary Dysplasia (BPD).

**Methods:**

Here we report a prospective cohort study of extremely preterm neonates wherein infants diagnosed with severe BPD expressed increased airway miR-219-5p and decreased platelet derived growth factor receptor alpha (PDGFR-α), a target of mir-219-5p and a key regulator of alveolarization, compared to post-conception age-matched term infants.

**Results:**

miR-219-5p was highly expressed in the pulmonary epithelial lining in lungs of infants with BPD by in situ* hybridization* of human infant lungs. In both in vitro and in vivo (mouse) models of BPD, miR-219-5p was increased on exposure to hyperoxia compared with the normoxia control, with a complementary decrease of PDGFR-α. To further confirm the target relationship between miR‐219 and PDGFR-α, pulmonary epithelial cells (MLE12) and lung primary fibroblasts were treated with a mimic of miR-219-5p and a locked nucleic acid (LNA) based inhibitor of miR-219-5p. In comparison with the control group, the level of miR‐219 increased significantly after miR‐219 mimic treatment, while the level of PDGFR-α declined markedly. LNA exposure increased PDGFR-α. Moreover, in BPD mouse model, over-expression of miR-219-5p inhibited alveolar development, indicated by larger alveolar spaces accompanied by reduced septation.

**Conclusions:**

Taken together, our results demonstrate that increased miR-219-5p contributes to the pathogenesis of BPD by targeting and reducing PDGFR-α. The use of specific miRNA antagonists may be a therapeutic strategy for preventing the development of BPD.

## Background

Bronchopulmonary dysplasia (BPD) is a chronic lung disease with a high mortality rate that primarily affects premature infants receiving prolonged oxygen supplementation or mechanical ventilation [1]. The pathogenesis of this disease involves impaired alveolar septation accompanied by abnormal vascular remodeling, lung inflammation, and fibrosis [2]. The critical regulatory pathways involved in normal lung development depend upon integration of numerous signals from multiple (e.g. Wnt, TGF-β, Hedgehog, Retinoid) cross-talking pathways [3, 4]. Previously, our group has identified dynamic microRNA-regulated interaction networks involved in normal lung development [5].

MicroRNAs (miRNAs) are a large conserved class of short noncoding RNA that usually function at the post-transcriptional level in diverse biological pathways [6]. One single miRNA has the potential to bind target sequences in multiple messenger RNAs (mRNAs). miRNAs have the ability to interact with the 3′ untranslated region of mRNAs to induce mRNA degradation and translational repression. However, miRNAs can also activate translation or regulate transcription [6]. The interactions with the target gene are dynamic and diverse. The aberrant expression of different miRNA has been recognized in several lung diseases including BPD and has been reported extensively by our group [7–10] and others [11].

In an integrated genomic analysis conducted using the DNA repository of the Eunice Kennedy Shriver National Institute of Child Health and Human Development Neonatal Research Network (NICHD NRN), BPD lung tissue expressed multiple dysregulated genes within the miR-219 pathway [8]. Using online bioinformatics tools (DIANA LAB, PicTar, miRDB, Targetscan and miRanda, etc.), we and other researchers found that platelet derived growth factor receptor alpha PDGFR-α was a top predicted target of miR-219-5p [12]. miR-219-5p has also been implicated in neurodegeneration involved in Alzheimer’s disease and primary age related tauopathy as it regulates tau toxicity and expression at the post-transcriptional level. In addition, miR-219 has been discovered to be a downstream regulator of neural stem cell as well as oligodendrocyte proliferation and differentiation in mammalian brains through modulating platelet-derived growth factor receptor alpha (PDGFR-α) expression, which has also been shown to be vital for normal lung development and formation of alveoli [13–16]. In the saccular and alveolar stage of lung development, PDGFR-α-expressing cells differentiate into α-smooth muscle actin- and elastin-producing myofibroblasts, forming the secondary alveolar crests that are required for alveologenesis [16, 17].

Based on the existing literature, we hypothesized that increased miR-219-5p modulates inhibition of alveolar septation in BPD via reductions of PDGFR-α. To better understand the role of miR-219-5p and its targets in normal and abnormal alveolar septation, we conducted a prospective cohort study to analyze the expression of miR-219-5p and subsequently of its target PDGFR-α in healthy infants compared to infants with severe BPD. To understand the underlying mechanisms of BPD pathogenesis, we evaluated the effects of inhibition and over-expression of miR-219-5p in vitro in primary lung fibroblasts and MLE12, as well as in a murine model of BPD.

## Materials and methods

### Human tracheal aspirate samples

A prospective cohort study of extremely preterm infants (less than or equal to 28 week’s gestation) and post-conception age matched full-term infants was conducted at University of Alabama at Birmingham, AL between October 2014 and December 2018. Tracheal aspirate (TA) samples were collected from preterm infants with established severe BPD at 36 week’s postmenstrual age (PMA) and from full term infants who were 37–38 week’s gestation (total *n* = 60 infants, 30 in each group). Severe BPD (requiring > 30% O_2_ or continuous positive airway pressure (CPAP)/mechanical ventilation) at 36 weeks PMA was characterized using the physiologic definition [18]. The gestational age–matched full-term infants were intubated at or within 6 h of birth due to either surgical indication (congenital heart disease, abdominal wall defect) or perinatal depression (with no signs of meconium aspiration syndrome or other lung pathology), and did not have any lung disease. TA specimens were obtained from infants as clinically indicated, per unit protocol, after ensuring that the infant was adequately oxygenated. The protocol for TA collection involved instillation of 1 ml sterile isotonic saline into the infant’s endotracheal tube, manual bagging through the endotracheal tube for 3 breaths, and suctioning of the fluid into a sterile mucus trap. The sample was separated into supernatant and cell pellet after centrifugation (3000*g* for 10 min). Samples were stored frozen in − 80 °C until further processing. The supernatant was used for analysis. miR 219-5p was detected by RT-PCR as described below (primer from Thermo Fisher Scientific hsa-miR-219 Cat number: 4427975 Assay ID 000522). PDGFR-α mRNA was detected by RT PCR (primer from Thermo Fisher Scientific Cat number: 4331182 Assay ID: Hs00998018_m1) and protein was detected by ELISA using a human ELISA kit from Thermo Fisher Scientific.

### Human tissue in situ hybridization

Human lung tissues were obtained at autopsy from preterm infants who died due to BPD at 36–44 weeks’ post-menstrual age (n = 4/group) and term stillborn infants (n = 4/group). In situ* hybridization* for miR 219-5p expression was performed on human autopsy tissues as previously described [19]. In brief, tagged probes specific for miR-219-5p were utilized. The probe/target complex was visualized after reaction of the alkaline phosphatase-linked conjugate with the chromogen (nitroblue tetrazolium and bromochloroindolyl phosphate), using a nuclear fast red counterstain. Negative controls included omission of the probe and the use of a scrambled probe.

### Cell culture and treatment

Murine lung epithelial ((MLE-12, ATCC CRL-2110)) and lung primary fibroblasts isolated by explant culture as described previously [20] were maintained in serum and antibiotic-free media (DMEM media). Cells were seeded in 12-well cell culture plates overnight and then were incubated at 85% O_2_ (hyperoxia) or 21% O_2_ (normoxia)in a specially designed hyperoxia cell culture chamber. There were approximately 2 × 10^5^ cells per well, which were subsequently collected 24 h later. miR-219-5p and PDGFR-α mRNA were measured by RT-PCR. For gain of miR-219-5p function analysis, cells were transfected with 15 pmol mirVana-hsa-miR-219-5p mimic or a negative control (Ambion) using Lipofectamine 2000 transfection reagents (Invitrogen) according to manufacturer’s protocol. At 48 h p.t., cells were collected for measurement of miR-219-5p and PDGFR-α mRNA by RT-PCR. For loss of miR-219-5p function analysis, LNA™ microRNA technology was employed to carry out in vitro miR-219-5p knockdown [21, 22]. LNA™ oligonucleotides have enhanced affinities for their targets as compared to regular DNA or RNA based oligonucleotides [21, 22]. The LNA™ for mmu-miR-219-5p (Ambion) is 21-nucleotides long and contains the following sequence: 5′-UGAUUGUCCAAACGCAAUUCU-3′. Cells were transfected with 50 nmoles. LNA miR-219-5p inhibitor or a negative control (Qiagen) using Lipofectamine 2000 transfection reagents (Invitrogen) according to the manufacturer’s protocol. At 48 h p.t., cells were collected for measurement of miR-219-5p (primer from Thermo Fisher Scientific hsa-miR-219 Cat number: 4427975 Assay ID 000522) and PDGFR-α (primer from Thermo Fisher Scientific Cat number: 4331182 Assay ID: Hs00998018_m1) mRNA by RT-PCR.

### Mouse studies

All animal procedures were approved by the Institutional Animal Care and Use Committee (IACUC) and were consistent with the Public Health Service policy on Humane Care and Use of Laboratory Animals (Office of Laboratory Animal Welfare, 2002). The use of human samples was approved by the Institutional Review Board (IRB). Total RNA were extracted from whole lung homogenates of newborn C57BL/6 J mice using miRNeasy Mini Kit from Qiagen at three developmental time points [postnatal day 1 (P1), P14, and P42]. miR-219-5p and PDGFR-α mRNA were measured by RT-PCR. All experiments were done with a minimum of six mice from at least two litters for each condition.

### Neonatal hyperoxia exposure model

Newborn C57BL/6 J mice exposed to normoxia (21% O_2_; control group) or hyperoxia (85% O_2_) in a sealed Plexiglas chamber with continuous oxygen monitoring, as previously described [23]. At P14, mice lung tissue was collected. MiR-219-5p and PDGFR-α mRNA were measured by RT-PCR and Western Blot.

### In vivo miR-219-5p mimic administration

For gain of function of miR-219-5p experiment, custom built mirVana-mmu-miR-219-5p mimic or a negative control from Thermo Fisher scientific was administered intranasally on P3, P7 and P10 (7 µg/g per mice dissolved in 5 μL molecular grade water) to mice. Our group has previously described these methods in detail [9, 10].

### Real-time RT-PCR

Total RNA from homogenized lung was extracted using TRIzol (Invitrogen, Carlsbad, CA), and reverse transcribed using the SuperScript III First-Strand Synthesis System for RT-PCR (Invitrogen). RT-PCR for PDGFR-α (primer from Thermo Fisher Scientific Cat number: 4331182 Assay ID: Hs00998018_m1) and miR-219-5p (primer from Thermo Fisher Scientific hsa-miR-219 Cat number: 4427975 Assay ID 000522) on homogenized lung were performed as described previously [23] using specific primers. Briefly, for RT of mature miRNA, 100 ng of DNase-treated total RNA was reverse transcribed using 100 U of SuperScript III Reverse Transcriptase enzyme and 100 nM of miR-219-5p stem-loop oligonucleotides [24]. The cDNA samples were then used for real-time PCR using miRNA-specific primers for miR-219-5p. Real-time PCR was performed on the MyiQ™ Single-Color Real-Time PCR detection System (Bio-Rad) using SYBR Green PCR Master Mix (Applied Biosystems). Two microliters of each cDNA sample were subjected to real-time PCR in a total volume of 20 µl using 100 nM of each primer. Thermocycling of miRNA cDNA was performing after 10 min of initial denaturation at 95 °C followed by 50 cycles of 15-s at 95 °C and 1 min annealing/extension at 60 °C. miR-219-5p expression levels were normalized to U6 snRNA. Expression levels of genes were normalized to 18S rRNA.

### Lung morphometry

Lung alveolar morphometry was performed as described previously [9]. Measurements of inflation-fixed lung sections for radial alveolar counts (RAC) were performed by an observer masked to sample identity [25].

### Western blot

Mouse lungs were homogenized in a tissue protein extraction reagent (Pierce Biotechnology, Rockford, IL) plus proteinase inhibitors (Roche Diagnostics, Basel, Switzerland), centrifuged at 12,000*g* for 15 min, and supernatant frozen at − 80 °C. Protein concentrations were measured by Bio-Rad Bradford protein assay (Bio-Rad, Hercules, CA). Western blots were done as described previously [23]. The primary antibody was rabbit anti-PDGFR-α (1:1000; Abcam, ab203491) in TBST buffer overnight at 4 °C. The secondary antibody was a goat anti-rabbit horseradish peroxidase (HRP) secondary antibody (Abcam) used at 1:10,000 dilution for 2 h at room temperature.

### Statistical analysis

GraphPad version 7 was used for the analysis of our data. All results are expressed as means ± SE. Data were analyzed by paired two-tailed t-test. A P value of less than 0.05 was considered significant.

## Results

### miR-219-5p is increased whereas PDGFR-α is decreased in airways of preterm infants with severe BPD when compared with gestation matched controls

**Fig. 1:**
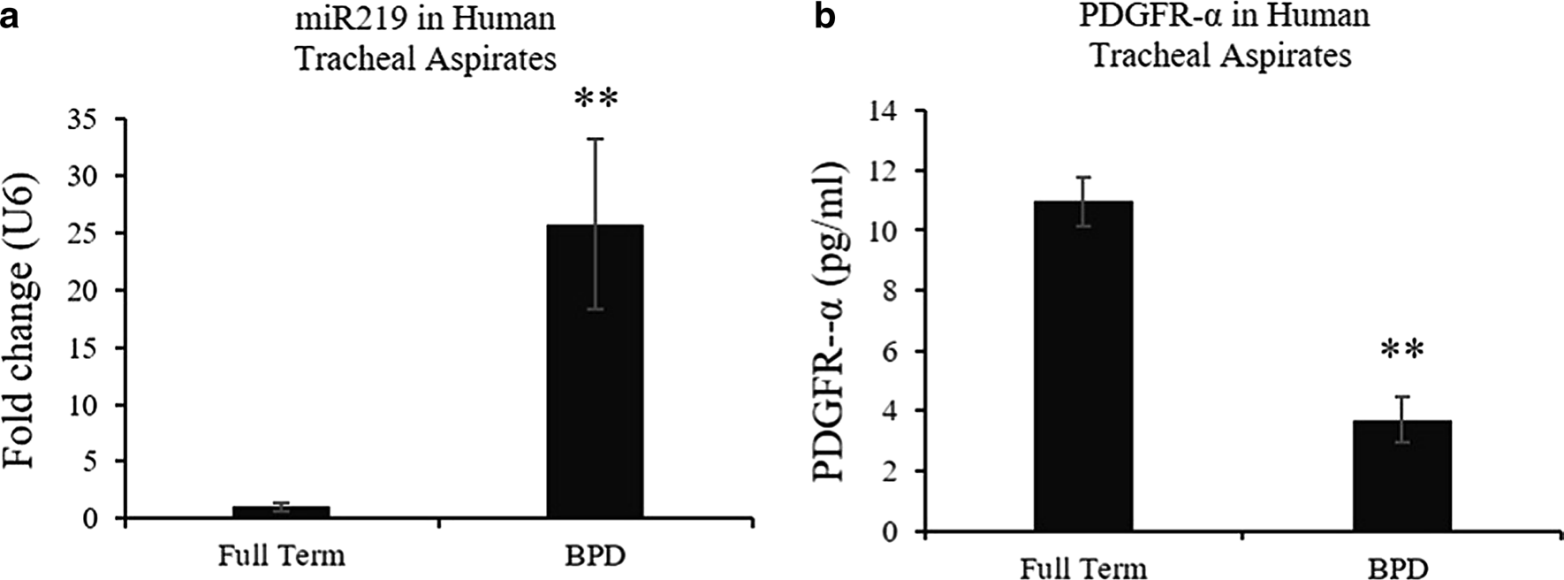
Airway miR-219-5p (miR 219) and PDGFR-α for BPD. 30 human tracheal aspirate samples collected from full term and BPD infants were analyzed for miR-219 (**a**) by qPCR and PDGFR-α (**b**) by ELISA. Values are means ± SE; *n* = 30 samples /group. *P ˂ 0.05 vs. corresponding group

We collected 30 tracheal aspirate samples from infants with established severe BPD and 30 tracheal aspirate samples from term controls (Total 60, *n* = 30 each group). Expression of miR-219-5p was markedly increased in tracheal aspirates of infants with severe BPD, compared with full term controls (Fig. 1a, P < 0.05). PDGFR-α mRNA expression was correspondingly reduced in infants with BPD compared with full-term (Fig. 1b, P < 0.05).

### miR-219-5p is highly expressed in severe BPD and predominantly in the pulmonary epithelial lining of lungs.

**Fig. 2:**
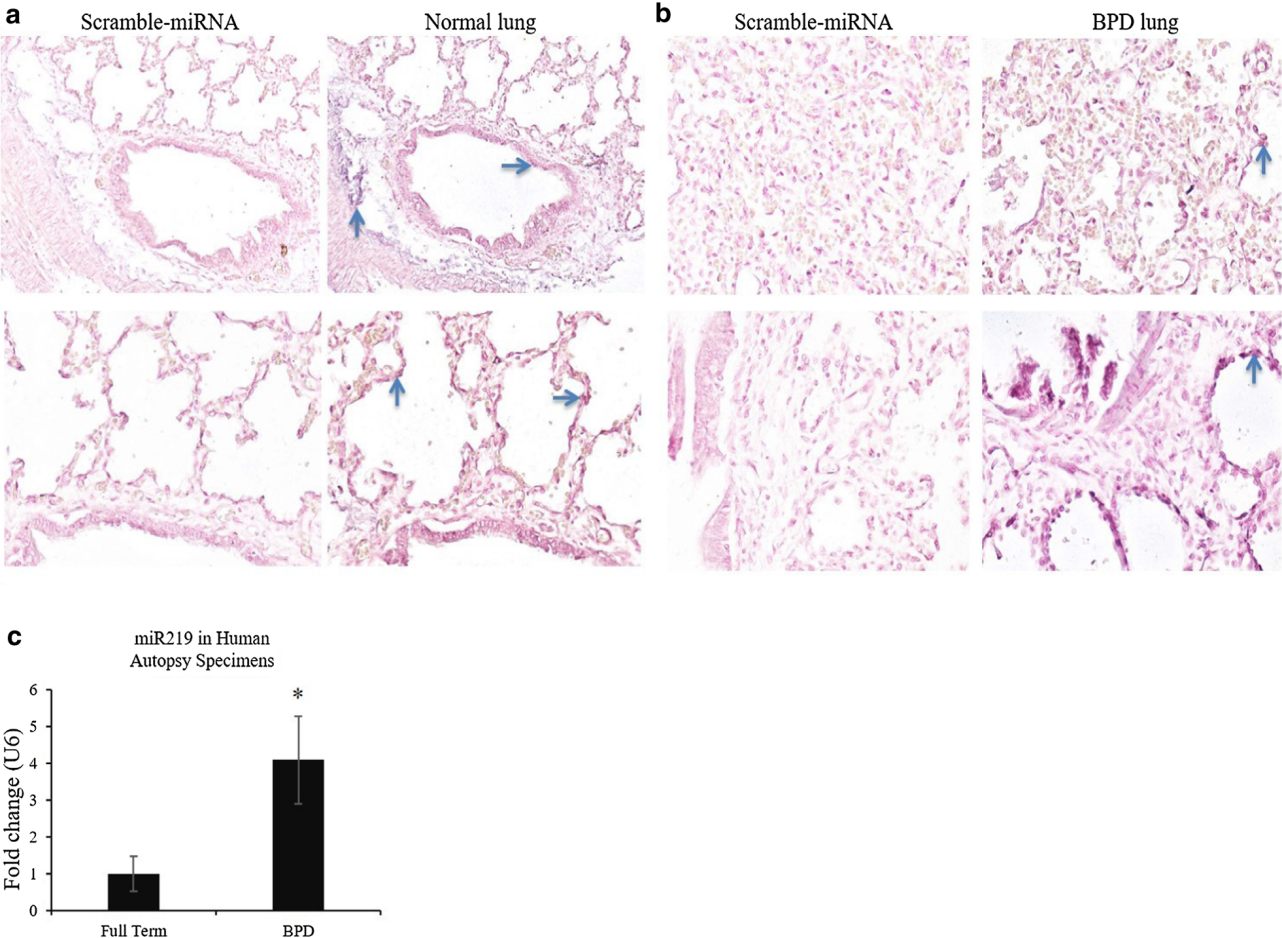
Expression of miR-219-5p (miR 219) is increased in human bronchopulmonary dysplasia (BPD), compared with term lung of comparable gestational age. **a**, **b***: *In situ* hybridization* of miR-219 in FFPE sections of human BPD and normal lungs. **c**: miR-219 expression by qPCR in RNA isolated from human lung samples of full term lung and BPD lung at 36–40 wk postmenstrual age (PMA). Values are means ± SE; *n* = 4 samples/group. *P ˂ 0.05 vs. other groups

To determine the level and location of miR-219-5p in human infant lungs, we used in situ* hybridization* (ISH) of miR-219-5p in formalin-fixed paraffin-embedded (FFPE) sections of human lung tissues retrieved by autopsy. Scramble probes were used as negative control. The sample types represented normal tissue (n = 2) and tissue affected by BPD (n = 5). Expression of miR-219-5p was barely detectable in normal lung, as compared to BPD lungs (Fig. 2a and b). miR-219-5p staining was localized to the epithelial cells (bronchiolar lining cells) and not present in stromal cells in BPD lung (Fig. 2a and b). We also performed RT-PCR of miR-219-5p using RNA isolated from human lung tissues retrieved by autopsy. Expression of miR-219-5p was significantly increased in the lungs of infants with BPD compared with full term infants (Fold change over the expression of U6 snRNA) (Fig. 2c, n = *4,* P < 0.05). These results are in concurrence with our findings in infant tracheal aspirates.

### Hyperoxia increases miR-219-5p and decreases PDGFR-α expression in vitro

**Fig. 3:**
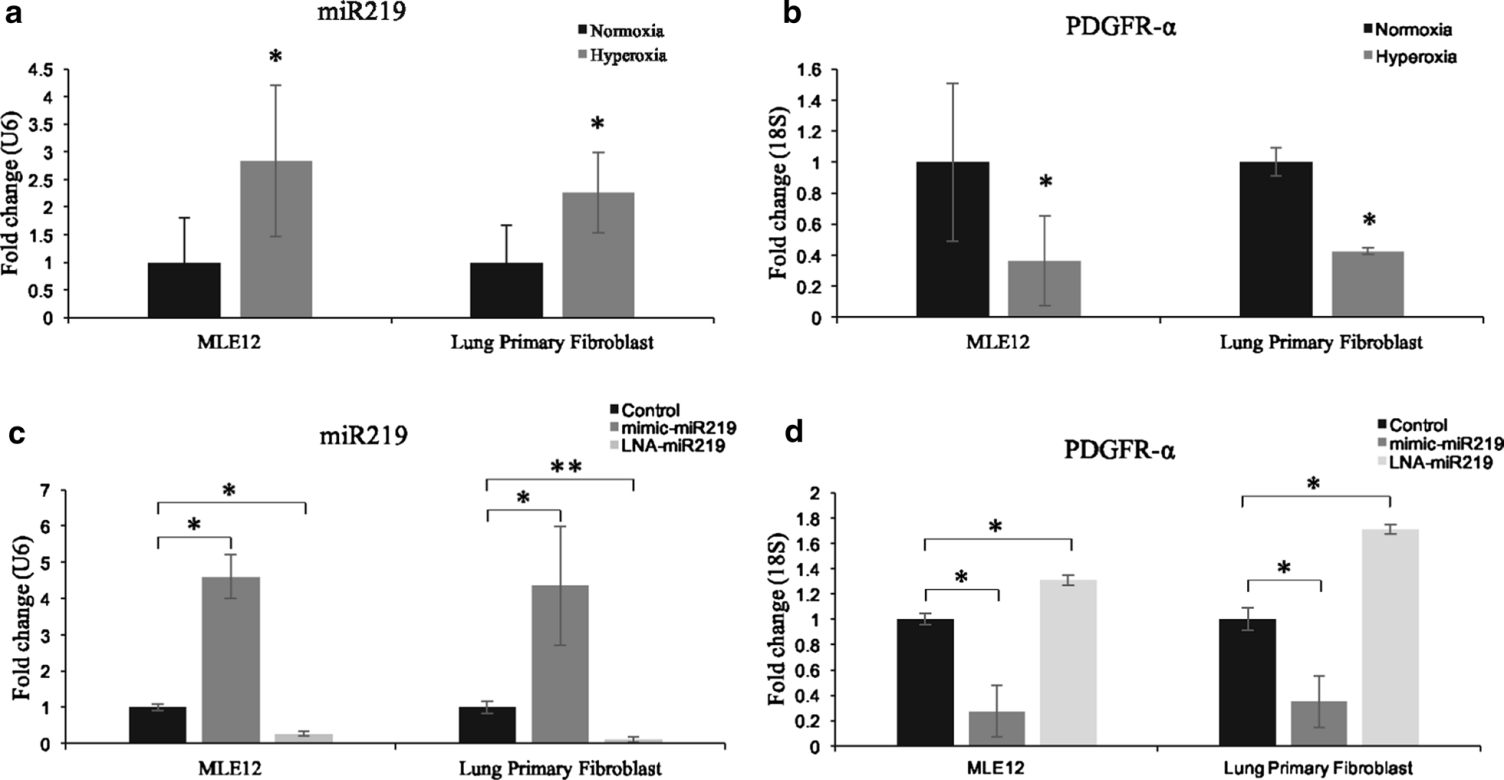
Expression of miR-219-5p (miR-219) and PDGFR-α in MLE12 and primary lung fibroblasts exposed to air or hyperoxia and treated with either mimic miR-219 or locked nucleic acid (LNA)-miR-219 inhibitor. **a**, **b**: miR-219 was increased while PDGFR-α was decreased in MLE12 and primary lung fibroblasts exposed to hyperoxia for 24 h, compared with normoxia. **c**, **d** Representative graphs showing significant decrease of PDGFR-α in mimic miR-219 transfected group and increase of PDGFR-α in LNA-miR-219 inhibitor transfected group compared to its control in both MLE12 and primary lung fibroblasts. Values are means ± SE; *P ˂ 0.05 vs. corresponding group

RT-PCR showed that miR-219-5p was primarily expressed in epithelial cells (Fig. 3a), which is consistent with our data obtained from ISH. Mouse lung epithelial cells (MLE 12) were exposed to normoxia or hyperoxia, and cells were collected after 24 h of exposure. Hyperoxia exposure increased miR-219-5p expression by approximately threefold as compared to normoxic control (Fig. 3a, P < 0.05). Conversely, PDGFR-α expression was downregulated significantly following hyperoxia exposure in MLE12 (Fig. 3b, P < 0.05). The same results were also confirmed in lung primary fibroblasts, where hyperoxia significantly increased miR-219-5p level and reduced the mRNA expression of PDGFR-α (Fig. 3a and b, P < 0.05).

### Inhibition of miR-219-5p using LNA-miR-219-5p increases PDGFR-α, while overexpressing of miR-219-5p using the mimic miR-219-5p decreases PDGFR-α in vitro

In MLE12 cells, treatment with the miR-219-5p mimic increased miR-219-5p expression up to fourfold (Fig. 3c, P < 0.05). In contrast the LNA inhibitor of miR-219-5p decreased miR-219-5p expression by about 80% (Fig. 3c, P < 0.05). The same results were achieved in the lung primary fibroblasts (Fig. 3d, P < 0.05). In addition, PDGFR-α expression following treatment with both mimic-miR-219-5p and LNA of miR-219-5p were tested in both MLE12 cells and lung primary fibroblasts. As expected, the miR-219-5p mimic attenuated PDGFR-α expression whereas the LNA miR-219-5p significantly increased PDGFR-α expression when compared with controls (Fig. 3d, P < 0.05). Collectively, these findings confirmed PDGFR-α as a downstream target of miR-219-5p and demonstrated that increased miR-219-5p plays an important role in BPD pathogenesis by reducing PDGFR-α expression.

### miR-219-5p and PDGFR-α expression level in mouse lungs at different stages of development

**Fig. 4:**
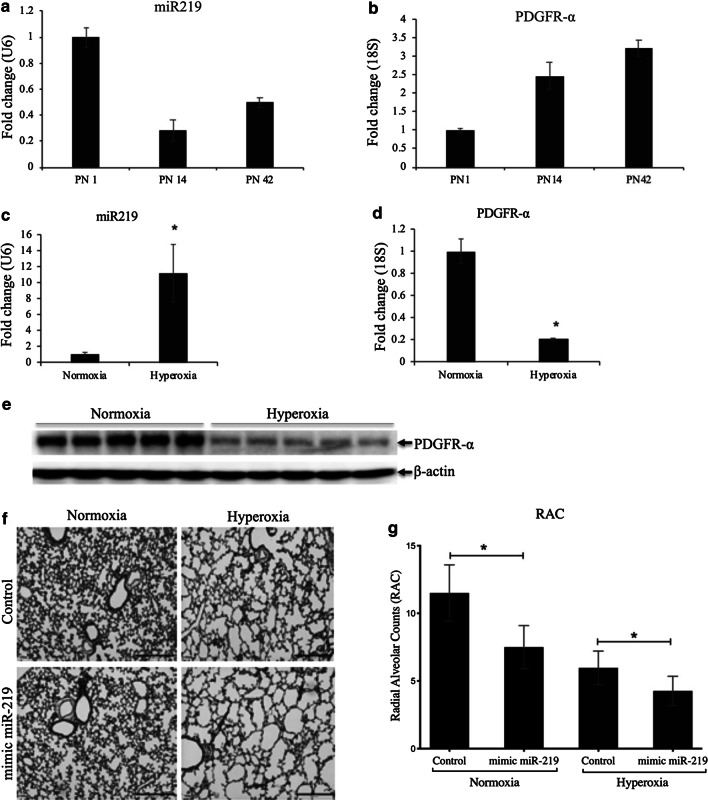
miR-219-5p (miR-219) and PDGFR-α during lung development and effect of hyperoxia and mimic miR-219 on miR-219 in a BPD mouse model. **a** Expression of miR-219 in lung homogenates from mice at 4, 7, and 14 days of age (P1, P14, and P42). **b** Expression of PDGFR-α by quantitative RT-PCR (qPCR) at P1, P14, and P42, showing increase PDGFR-α at P14, and P42 when miR-219 is decreased. Lung homogenates from mice at 14 days of age exposed to air or hyperoxia were analyzed by qPCR of RNA from lung homogenates for miR-219 (**c**) and PDGFR-α (**d**) or by western blot for PDGFR-α (**e**). **f** Representative photomicrographs of H&E-stained sections of alveolar regions from lungs of mice at 14 days of age administered either with mimic miR-219, or control mimic. **g** Radial alveolar counts (RAC) of mice administered control vs mimic miR-219 in normoxia and hyperoxia. Values are means ± SE; *n* = 6 mice/group. *P ˂ 0.05 vs. corresponding group

We monitored the expression level of miR-219-5p through qPCR with RNA from mouse lungs at different stages of development (P1, P14, and P42). The mice exhibited significantly decreased levels of miR-219-5p on day 14 and day 42 (vs. day1) during the course of alveolar septation (Fig. 4a, P < 0.05). Corresponding increase in PDGFR-α expression was seen from P1 to P42 (Fig. 4b, P < 0.05).

### Neonatal hyperoxia increases miR-219-5p and reduces PDGFR-α expression in vivo

In the mouse model of BPD, C57BL/6 J mice were exposed to hyperoxia after birth from P4 to P14. Air-vehicle mice were used as control. Following hyperoxia exposure, the miR-219-5p level in mice was significantly increased (Fig. 4c, P < 0.05). Conversely, the PDGFR-α expression was downregulated as shown by RT-PCR and Western blot (Fig. 4d and e), consistent with the results obtained in vitro.

### Gain of miR-219-5p function inhibited alveolar development in-vivo

To further explore the direct functional role of miR-219-5p in BPD, we performed in vivo gain of function experiments with a mimic of miR-219-5p. Over-expression of miR-219-5p using the miR-219-5p mimic inhibited alveolar development following even in normoxic conditions. This was demonstrated by larger alveolar spaces and reduced alveolar septation observed during histological examination. Further lung morphometric analysis was carried out by estimating the radial alveolar counts, which was reduced (vs. control animals) that were treated with the miR-219-5p mimic both in normoxia and hyperoxia (Fig. 4f and g, n = 6, P < 0.05). These morphological and phenotypical changes were similar to that observed during the progression of BPD, which is attributed to low levels of PDGFR-α production that occurs in dysfunctional lung development. These data indicate that miR-219-5p may play a critical role in BPD pathogenesis.

## Discussion

In the present study, we have obtained direct evidence for the role of miR-219-5p in the pathogenesis of BPD through targeting PDGFR-α. Multiple previous studies have explored PDGFR-α as a target of miR-219. In oligodendrocyte differentiation and proliferation, PDGFR-α was determined to be directly regulated by miR-219 [14]. In addition, PDGFR-α was also found to be expressed in mammalian neural stem cells, where it has also been discovered to be directly inhibited by miR219 [14]. Prior studies have also shown that miR219-5p may function as a tumor suppressor by targeting PDGFR-α in colorectal cancer [12]. It has also been shown to inhibit receptor tyrosine kinase pathways through targeting EGFR in glioblastoma [26]. Previous studies have also suggested that microRNAs and PDGFR-α play a role in pulmonary morphogenesis and lung injury. Our current study extends this work to another miRNA and its possible target which is critical for alveolar development.

Initially in our prospective human study, we found that infants with BPD have a higher expression of miR-219-5p and a lower expression of PDGFR-α in tracheal aspirates. The increase of miR-219-5p expression was further verified in human lung epithelial cells as demonstrated by in situ* hybridization*. Subsequently, we confirmed these findings both in vitro hyperoxia models of BPD and in newborn mouse BPD models. Hyperoxia significantly increased miR-219-5p level and reduces the mRNA expression of PDGFR-α in both in vitro and in vivo mouse models. Most importantly the over-expression of miR-219-5p in mice inhibited alveolar development as evidenced by larger alveolar spaces in both normoxia and hyperoxia, thus explaining the direct causative role of miR-219-5p in hyperoxia induced lung injury.

Our results are consistent with prior studies that have explored PDGF signaling and the essential role that it plays in embryonic and postnatal lung development [27]. The over-expression of miR-219-5p and subsequently reduced expression of PDGFR-α resulted in deranged lung development in both human and murine models of BPD. Multiple previous studies have attempted to characterize the expression pattern of PDGFR-α in addition to its ligands, PDGF-A and –C, within the lung tissue [27–29]. In murine lungs examined at different stages of development, PDGFR-α was found to be widely expressed in the mesenchyme in embryonic lungs, while its ligand PDGF-A expression was localized to the lung epithelium. Postnatally, PDGFR-α expression was associated with increased expression of α-SMA, which suggests that PDGF-A activates PDGFR-α in myofibroblast precursors. This interaction produces myofibroblast differentiation, migration, and remodeling of the cytoskeleton that is necessary for proper alveolar development and septation. In addition, PDGFR-α is responsible for Type 2 alveolar epithelial cell differentiation and self-renewal [27–29]. Reduced expression of PDGFR-α results in failure of alveolar septation and the formation of dilated distal airways, which is characteristic of BPD and consistent with our findings [28].

In our study, we found that increased expression of miR219-5p was associated with decreased expression of PDGFR-α in both in vitro and in vivo models. Based on prior studies, miR219-5p regulates the expression of PDGFR-α as well as other receptors in the tyrosine kinase pathway via complete or partial complementary sequences at the 3′ untranslated region of the gene thus causing reduction of protein translation or degradation of mRNA [6, 12, 14, 26]. Based on the results of our study, we speculate that miR219-5p results in suppression of PDGFR-α expression within the developing lung, which is associated with decreased alveolar septation and increased alveolar size. These results suggest miR219-5p plays a role in the pathogenesis of BPD through an interaction with PDGFR- α.

## Conclusion

The major strength of our study is the validation of novel signals in human infants, in addition to in vitro and in vivo model of BPD. Our results demonstrate that miR-219-5p plays an important role in the pathogenesis of BPD by targeting PDGFR-α. Our data not only reveals the strong biomarker potential of miR-219-5p for predicting the development of severe BPD in preterm infants, but also lays the foundation for future therapeutics development based on miR-219-5p antagonism. Furthermore, PDGFR-α has been shown to be expressed in both the pulmonary artery smooth muscle cells (PASMC) and the endothelial cells [30]. Severe BPD is marked by significant pulmonary hypertension in human and mice, hence elevating PDGFR-α signaling could prove beneficial in BPD induced pulmonary hypertension in preterm infants. Mir-219-5p antagonism in the murine model is currently a part of other ongoing studies, which if positive will lead to similar validation studies in large animals. Overall, the results from presented studies advance our understanding of miR-219-5p, as well as identify new targets for preventing BPD.

## Data Availability

Not applicable.

## Abbreviations

miR: MicroRNA
mRNAs: Messenger RNAs
BPD: Bronchopulmonary Dysplasia
NICHD NRN: National Institute of Child Health and Human Development Neonatal Research Network
TA: Tracheal aspirate
CPAP: Continuous positive airway pressure
ISH: in situ* hybridization*
FFPE: Formalin-fixed paraffin-embedded
